# Long-term exposure to air pollution and lung function among children in China: Association and effect modification

**DOI:** 10.3389/fpubh.2022.988242

**Published:** 2022-12-14

**Authors:** Jingjing Teng, Jie Li, Tongjin Yang, Jie Cui, Xin Xia, Guoping Chen, Siyu Zheng, Junhui Bao, Ting Wang, Meili Shen, Xiao Zhang, Can Meng, Zhiqiang Wang, Tongjun Wu, Yanlong Xu, Yan Wang, Gang Ding, Huawei Duan, Weidong Li

**Affiliations:** ^1^Anhui Center for Disease Control and Prevention, Public Health Research Institute of Anhui Province, Hefei, China; ^2^Department of Occupational and Environmental Health, School of Public Health, Capital Medical University, Beijing, China; ^3^Beijing Key Laboratory of Environmental Toxicology, School of Public Health, Capital Medical University, Beijing, China; ^4^Chinese Center for Disease Control and Prevention, National Institute for Occupational Health and Poison Control, Beijing, China; ^5^National Center for Chronic and Non-communicable Disease Control and Prevention, Chinese Center for Disease Control and Prevention, Beijing, China

**Keywords:** air pollution, long-term, lung function, children, effect modification

## Abstract

**Background:**

Children are vulnerable to the respiratory effects of air pollution, and their lung function has been associated with long-term exposure to low air pollution level in developed countries. However, the impact of contemporary air pollution level in developing countries as a result of recent efforts to improve air quality on children's lung function is less understood.

**Methods:**

We obtained a cross-sectional sample of 617 schoolchildren living in three differently polluted areas in Anhui province, China. 2-year average concentrations of air pollutants at the year of spirometry and the previous year (2017–2018) obtained from district-level air monitoring stations were used to characterize long-term exposure. Forced vital capacity (FVC), forced expiratory volume in 1 second (FEV_1_), and forced expiratory flow between 25 and 75% of FVC (FEF_25−75_) were determined under strict quality control. Multivariable regression was employed to evaluate the associations between air pollution level and lung function parameters, overall and by demographic characteristics, lifestyle, and vitamin D that was determined by liquid chromatography tandem mass spectrometry.

**Results:**

Mean concentration of fine particulate matter was 44.7 μg/m^3^, which is slightly above the interim target 1 standard of the World Health Organization. After adjusting for confounders, FVC, FEV_1_, and FEF_25−75_ showed inverse trends with increasing air pollution levels, with children in high exposure group exhibiting 87.9 [95% confidence interval (CI): 9.5, 166.4] mL decrement in FEV_1_ and 195.3 (95% CI: 30.5, 360.1) mL/s decrement in FEF_25−75_ compared with those in low exposure group. Additionally, the above negative associations were more pronounced among those who were younger, girls, not exposed to secondhand smoke, non-overweight, physically inactive, or vitamin D deficient.

**Conclusions:**

Our study suggests that long-term exposure to relatively high air pollution was associated with impaired lung function in children. More stringent pollution control measures and intervention strategies accounting for effect modification are needed for vulnerable populations in China and other developing countries.

## Introduction

Air pollution is ubiquitous and poses a significant threat to global health. Ambient pollutants get into the body mainly through breathing, with the potential of irritating airways and penetrating deeply into the lung, making respiratory system the primary target of adverse effects. According to the Global Burden of Diseases Study 2019, air pollution ranked second among risk factors contributing to chronic respiratory diseases (1). As children have a higher ventilation rate and usually spend more time outdoors than adults, they are more prone to air pollutants-induced respiratory problems. Lung function is an objective measure of respiratory health and reduced lung function during childhood has clinical relevance with lung growth and later respiratory morbidity and mortality (2). Thus, it is vital to study the impact of air pollution on the lung function of children.

Compared with short-term exposure, studying long-term exposure to air pollution has more implications because of greater effect and less reversibility of lung function. Both cross-sectional and longitudinal studies have shown that long-term exposure to air pollution may affect lung function among children. For example, a multicenter study taking advantage of data from several European birth cohorts found that the annual average concentrations of fine particulate matter (PM_2.5_) and nitrogen dioxide (NO_2_) at the current address were negatively associated with forced vital capacity (FVC) and forced expiratory volume in 1 second (FEV_1_) (3). The Dutch population-based Prevention and Incidence of Asthma and Mite Allergy cohort study demonstrated that exposure to all pollutants during the preschool period was associated with reduced growth of FEV_1_ (4). However, most of the previous studies were conducted in developed countries with typically low pollution levels (e.g., PM_2.5_ below 20 μg/m^3^). Indeed, more than 90% of global children were exposed to levels of air pollution above the World Health Organization (WHO) air quality guideline (5). China is the world's largest developing country where the air pollution level is much higher than that in developed countries. A few multicity studies carried out between 2009 and 2012 have linked long-term exposure to high level of air pollution to the impaired lung function of Chinese children (6, 7). To tackle this severe environmental issue and protect public health, the central government of China released a 5-year action plan in 2013, aiming at improving air quality and reducing heavy pollution days. With the implementation of supportive clean air actions, the population-weighted mean PM_2.5_ concentration was estimated to decrease from 61.8 μg/m^3^ in 2013 to 42.0 μg/m^3^ in 2017 (8). Despite the remarkable reduction, few studies have examined the association between long-term air pollution exposure and children's lung function in the context of moderate air pollution. Additionally, understanding factors that modify the respiratory effects of air pollution is crucial for screening high-risk subpopulations and designing targeted invention measures. Available investigations mainly focused on sex and asthma status (9), although studies simultaneously exploring the modification effects of demographic characteristics, lifestyle, and micronutrients are needed.

To address these knowledge gaps, we examined the associations of long-term exposure to air pollution with a range of lung function parameters and if there is an exposure-response relationship in 617 schoolchildren who resided in three differently polluted areas in a less developed province, China, between 2017 and 2018. Furthermore, whether the above associations varied by age, sex, secondhand smoke exposure, body mass index (BMI), physical exercise, and serum vitamin D were also tested.

## Methods

### Study population

Three districts were, respectively chosen from three cities located in Anhui province, central China, based on historical air monitoring quality data and hereafter referred to as low, moderate, and high polluted areas. A multistage cluster sampling strategy was used to recruit schoolchildren from these polluted areas. In the first stage, one primary school and one secondary school were randomly selected within each district. In the second stage, one class was randomly selected from grades 1–6 of primary school and grades 7–8 of secondary school. In the final stage, 26 children were randomly selected from each selected class. Children were eligible if they were aged 6–15 years, had lived in the current address for at least 2 years, and showed no respiratory symptoms in the previous 14 days before spirometry. A total of 620 schoolchildren participated in this study between October 2017 to May 2018. Among these, three children had incomplete data on the questionnaire, resulting in 617 children being included in the final analysis. With this sample size, we have 80% power to detect a Cohen's f of 0.126 considering a type I error of 5%.

### Data collection

According to the air quality data from environmental monitoring stations near to school, annual average concentrations of particulate matter with aerodynamic meter ≤10 μm (PM_10_), PM_2.5_, NO_2_, sulfur dioxide (SO_2_), ozone (O_3_), and carbon monoxide (CO) during 2017–2018 were calculated to characterize the long-term exposure levels of schoolchildren. In addition, 2-day (current day and 1 day before spirometry, lag01) moving average of PM_2.5_ concentration was calculated as a surrogate of short-term exposure.

Through face-to-face interviews, a standardized questionnaire was administered to all participants by experienced investigators to collect data on sociodemographic characteristics, lifestyle factors, self-reported chronic diseases, and medication history. Physical measurements including height and weight were also performed with participants in light clothing without shoes. Besides, 5 mL of venous blood and 50 mL of urine were obtained from each participant after an overnight fast of 8 h.

Secondhand smoke exposure was defined as exposure to passive tobacco smoking at least once per week. Children who got <180 min per week of moderate and/or vigorous activities were considered physically inactive. Vegetable and fruit intake was categorized as insufficient if less than four servings were consumed per day, otherwise as sufficient. Parental education was divided into two classes, high school or below and college or above. BMI was calculated by dividing weight (kg) by squares of height (m). Age- and sex-specific BMI z-score was further calculated based on WHO growth reference, and a BMI z-score equal to or above the corresponding 85th percentile was deemed as overweight/obesity (10).

### Measurement of lung function

Lung function tests were performed by two trained technicians following the guidelines of the American Thoracic Society and European Respiratory Society (11). The child, standing and wearing a nose clip, was asked to blow at least three times into MasterScreen Pneumo spirometer (CareFusion, Germany) with a disposable mouthpiece. Acceptable reproducibility was obtained if the differences between the two highest FVC values and FEV_1_ values were both ≤150 mL (or within 5%). For children whose lung function results did not meet this standard, a maximum of eight blows were required. An expert panel conducted quality control on all reports of lung function tests, and calibration was done daily using a 3L syringe. FVC, FEV_1_, and forced expiratory flow between 25 and 75% of FVC (FEF_25−75_) were derived from the best curve. FEV_1_/FVC is the ratio of FEV_1_ to FVC. Moreover, the predicted values of FVC, FEV_1_, and FEF_25−75_ were calculated based on the 2012 Global Lung Function Initiative reference equation taking age, sex, and height into account (12). Poor lung function was defined as observed FVC <85% predicted value, FEV_1_ <85% predicted value, FEF_25−75_ <75% predicted value, and FEV_1_/FVC<85% according to relevant cutoff values applicable to children (13, 14).

### Serum vitamin D detection

The serum was separated by centrifugation and stored at −80°C. Serum level of 25(OH)D (sum of 25(OH)D_2_ and D_3_) was detected by liquid chromatography tandem mass spectrometry (AB6500, U.S.). The reference standard was purchased from Sigma-Aldrich (U.S.) and other analytical grade chemical reagents were commercially available from Merck (Germany). In brief, 150 μL of serum was pipetted into a microtube and mixed with 15 μL internal standard (1,000 ng/mL). Then 150 μL aqueous zinc sulfate and 300 μL acetonitrile were added to precipitate protein, and the tube was vortexed for 30 s and left for 15 min at room temperature. After adding 750 μL hexane, the mixture was vortexed for 30 s and centrifugated at 13,000 rpm for 5 min. Following drying under nitrogen gas of 0.5 mL supernatant, the residue was reconstituted using 200 μL mobile phase for further analysis. All samples were performed in duplicate, and the coefficients of variation for intra- and inter-assay were <10%.

### Statistical analysis

Differences between exposure groups were tested using analysis of variance for continuous variables and chi-square test for categorical variables. Multivariable linear regression models were used to examine the associations between long-term air pollution exposure levels and lung function parameters. The model was adjusted for age (continuous), sex (boys/girls), height (continuous), weight (continuous), secondhand smoke exposure (yes/no), physical activity (active/inactive), vegetable and fruit intake (sufficient/insufficient), and parental education (high school or below/college or above). Similar to previous studies that accounted for the confounding effect of short-term exposure (15, 16), PM_2.5_ concentration on lag01 days of lung function measurement was also included as a covariate. When testing the linear trends of lung function parameters with increasing air pollution levels, the exposure group was modeled as an ordinal variable. For associations with the prevalence of poor lung function, multivariable logistic regression models were employed with the same covariates.

Effect modification of the above associations was further evaluated through subgroup analyses stratified by age, sex, secondhand smoke exposure, BMI, physical activity, and serum vitamin D. To this end, age was dichotomized as 11 years or younger and 12 or older, and vitamin D was also dichotomized with its median (i.e., 16.6 ng/mL) as a cutoff value. For a more straightforward comparison between subgroups, marginally adjusted means or probability was computed for all combinations of exposure group and stratified variable, which are weighted averages reflecting confounder distribution in the total population (17). To respect 0 and 1 bounds, the predicted probability and its confidence interval (CI) were first generated on the logit scale and then transformed back to the probability scale. The interaction was formally tested by introducing a product term between exposure group and modifier, and a Wald test evaluating the joint significance of all associated cross-product terms was used to assess statistical significance. Furthermore, the joint impacts of air pollution and secondhand smoke on lung function were investigated by classifying the participants into six groups according to air pollution levels and secondhand smoke exposure status, with schoolchildren in the low exposure group and not exposed to secondhand smoke as reference.

Several sensitivity analyses were conducted as follows: (a) using percent predicted values instead of observed values of lung function parameters as dependent variables; (b) excluding children exposed to secondhand smoke; (c) excluding children with a self-reported diagnosis of asthma; (d) additionally adjusted for short-term NO_2_ or O_3_.

All analyses were done with R (version 3.6.0) and a *P*-value < 0.05 was considered statistically significant. Mean differences and odds ratio (OR) and their 95% CI were reported for results of linear and logistic regression models, respectively.

## Results

Supplementary Figure S1 displays the geographic locations of six selected schools in Anhui province, China. The 2-year mean concentrations of PM_10_, PM_2.5_, NO_2_, SO_2_, O_3_, and CO were 72.4 μg/m^3^ [standard deviation (SD) = 42.7], 44.7 μg/m^3^ (SD = 33.3), 31.2 μg/m^3^ (SD = 19.0), 12.5 μg/m^3^ (SD = 5.6), 94.1 μg/m^3^ (SD = 45.9), and 0.8 mg/m^3^ (SD = 0.3), respectively (Table 1). While the concentrations of PM_10_, PM_2.5_, and O_3_ increased from low exposure group to high exposure group, the concentration of NO_2_ was highest in moderate group. Considering that NO_2_ is an indicator of vehicle exhaust, this discrepancy may be explained by more urbanization occurring in the district where the moderate group was recruited.

**Table 1:** Air pollutant concentrations, general characteristics, and lung function parameters among schoolchildren, overall and by exposure group.

	**Total (*n* = 617)**	**Exposure group**			***P*-value**
		**Low (*n* = 209)**	**Moderate (*n* = 206)**	**High (*n* = 202)**	
**Air pollutant**					
PM_10_ (μg/m^3^)	72.4 ± 42.7	46.6 ± 24.8	75.3 ± 37.8	95.3 ± 47.1	<0.001^c^
PM_2.5_ (μg/m^3^)	44.7 ± 33.3	24.9 ± 17.9	51.5 ± 32.9	57.9 ± 36.2	<0.001^c^
NO_2_ (μg/m^3^)	31.2 ± 19.0	17.7 ± 5.3	46.7 ± 20.9	29.3 ± 13.8	<0.001^c^
SO_2_ (μg/m^3^)	12.5 ± 5.6	11.4 ± 3.1	9.7 ± 4.7	16.5 ± 6.0	<0.001^c^
O_3_ (μg/m^3^)	94.1 ± 45.9	73.1 ± 26.5	100.5 ± 48.7	108.8 ± 50.5	<0.001^c^
CO (mg/m^3^)	0.8 ± 0.3	0.7 ± 0.2	0.9 ± 0.3	0.8 ± 0.3	<0.001^c^
**Characteristics**					
Age (years)	10.6 ± 2.2	10.3 ± 2.3	10.8 ± 2.1	10.6 ± 2.3	0.089^c^
**Sex (** ***n*** **, %)**					
Boys	311 (50.4)	105 (50.2)	103 (50.0)	103 (51.0)	0.979^d^
Girls	306 (49.6)	104 (49.8)	103 (50.0)	99 (49.0)	
**Secondhand smoke exposure (** ***n*** **, %)**					
No	474 (76.8)	142 (67.9)	151 (73.3)	181 (89.6)	<0.001^d^
Yes	143 (23.2)	67 (32.1)	55 (26.7)	21 (10.4)	
**Physical activity (** ***n*** **, %)** **^a^**					
Inactive	336 (56.1)	105 (52.8)	137 (67.8)	94 (47.5)	<0.001^d^
Active	263 (43.9)	94 (47.2)	65 (32.2)	104 (52.5)	
**Vegetable and fruit intake (** ***n*** **, %)**					
Insufficient	299 (48.5)	107 (51.2)	77 (37.4)	115 (56.9)	<0.001^d^
Sufficient	318 (51.5)	102 (48.8)	129 (62.6)	87 (43.1)	
**Parental education (** ***n*** **, %)** **^b^**					
High school or below	426 (69.8)	61 (30.2)	179 (86.9)	186 (92.1)	<0.001^d^
College or above	184 (30.2)	141 (69.8)	26 (13.1)	16 (7.9)	
Height (cm)	143.8 ± 14.0	142.6 ± 14.4	143.1 ± 12.3	145.8 ± 15.0	0.048^c^
Weight (kg)	37.0 ± 12.1	35.1 ± 11.8	36.6 ± 10.9	39.4 ± 13.1	<0.001^c^
BMI (kg/m^2^)	17.4 ± 3.1	16.8 ± 3.0	17.5 ± 3.0	18.0 ± 3.2	<0.001^c^
BMI z-score	0.0 ± 1.1	−0.3 ± 1.3	0.0 ± 1.1	0.2 ± 1.2	<0.001^c^
Overweight/Obesity (*n*, %)	104 (16.9)	33 (15.8)	32 (15.5)	39 (19.3)	0.524^d^
Serum vitamin D (ng/mL)	17.5 ± 3.8	17.7 ± 3.8	16.5 ± 3.8	18.3 ± 3.3	<0.001^c^
**Lung function parameters**					
FVC (mL)	2384.8 ± 743.7	2426.9 ± 770.4	2280.3 ± 660.2	2447.8 ± 787.0	0.045^c^
FEV_1_ (mL)	2075.5 ± 636.0	2090.3 ± 639.2	2033.3 ± 581.0	2103.0 ± 685.2	0.498^c^
FEF_25−75_ (mL/s)	2451.6 ± 826.1	2417.5 ± 797.9	2535.9 ± 784.6	2400.9 ± 891.0	0.196^c^
FEV_1_/FVC (%)	87.4 ± 6.8	86.5 ± 6.4	89.6 ± 6.1	86.1 ± 7.4	<0.001^c^
Percent predicted FVC (%)	97.0 ± 13.0	101.1 ± 11.9	93.7 ± 13.0	96.1 ± 13.1	<0.001^c^
Percent predicted FEV_1_ (%)	95.9 ± 12.8	98.8 ± 11.4	94.9 ± 13.0	93.8 ± 13.5	<0.001^c^
Percent predicted FEF_25−75_ (%)	96.6 ± 23.1	97.3 ± 21.7	100.6 ± 23.4	91.9 ± 23.7	<0.001^c^
Percent predicted FEV_1_/FVC (%)	98.6 ± 7.6	97.5 ± 7.1	101.0 ± 6.8	97.3 ± 8.3	<0.001^c^
Low FVC (< 85% predicted, %)	91 (14.7)	15 (7.2)	43 (20.9)	33 (16.3)	<0.001^d^
Low FEV_1_ (< 85% predicted, %)	107 (17.3)	24 (11.5)	42 (20.4)	41 (20.3)	0.023^d^
Low FEF_25−75_ (< 75% predicted, %)	99 (16.0)	29 (13.9)	22 (10.7)	48 (23.8)	<0.001^d^
Low FEV_1_/FVC (< 85%, %)	204 (33.1)	72 (34.5)	48 (23.3)	84 (41.6)	<0.001^d^

The statistics are shown as mean ± SD or counts (percentages).

a 18 missing values for physical activity;

b 7 missing values for parental education;

c Analysis of variance;

d Chi-square test.

The general characteristics and lung function levels of schoolchildren are shown in Table 1. Although there were no significant differences across exposure groups in terms of age and sex, lower proportion of secondhand smoke exposure and parental education level, and higher BMI were observed for children with higher exposure levels. The averages of lung function parameters were 2384.8 mL (SD = 743.7) for FVC, 2075.5 mL (SD = 636.0) for FEV_1_, 2451.6 mL/s (SD = 826.1) for FEF_25−75_, and 87.4% (SD = 6.8) for FEV_1_/FVC. The prevalence of poor lung function for FVC, FEV_1_, FEF_25−75_, and FEV_1_/FVC was 14.7, 17.3, 16.0, and 33.1%, respectively.

The associations between air pollution exposure and lung function parameters are presented in Table 2. After adjusting for covariates, FVC, FEV_1_, and FEF_25−75_ showed inverse trends with increasing air pollution levels (p_trend_ = 0.030, 0.004, 0.012, respectively). Specifically, children in the high exposure group exhibited 73.3 (95% CI: −14.6, 161.2) mL decrement in FVC, 87.9 (95% CI: 9.5, 166.4) mL decrement in FEV_1_, and 195.3 (95% CI: 30.5, 360.1) mL/s decrement in FEF_25−75_ compared with those in the low exposure group. FEV_1_/FVC, reflecting the association patterns with FEV_1_ and FVC, was not significantly different between groups. As for associations with the prevalence of poor lung function, the results were generally insignificant regardless of the increased odds ratio in the high exposure group.

**Table 2:** Multivariable adjusted^a^ associations between air pollution levels and lung function parameters among all schoolchildren.

	**Estimate (95% CI)**	***P*-value^a^**	** $P_{\text{tren}{\text{d}}^{\text{a}}}$ **
**Continuous outcomes**			
**FVC**			
Low exposure	Ref	–	0.030
Moderate exposure	−8.5 (−165.3, 148.3)	0.915	
High exposure	−73.3 (−161.2, 14.6)	0.102	
**FEV**_**1**_			
Low exposure	Ref	–	0.004
Moderate exposure	−17.1 (−156.9, 122.8)	0.811	
High exposure	−87.9 (−166.4, −9.5)	0.028	
**FEF**_**25 − 75**_			
Low exposure	Ref	–	0.012
Moderate exposure	−140.2 (−434.2, 153.8)	0.349	
High exposure	−195.3 (−360.1, −30.5)	0.020	
**FEV**_**1**_ **/FVC**			
Low exposure	Ref	–	0.109
Moderate exposure	0.0 (−3.5, 3.4)	0.991	
High exposure	−1.1 (−3.1, 0.8)	0.240	
**Binary outcomes**			
Low FVC (< 85% predicted)			
Low exposure	Ref	–	0.124
Moderate exposure	1.22 (0.26, 5.63)	0.803	
High exposure	1.79 (0.72, 4.54)	0.213	
Low FEV_1_ (< 85% predicted)			
Low exposure	Ref	–	0.122
Moderate exposure	1.34 (0.31, 5.62)	0.687	
High exposure	1.74 (0.77, 3.96)	0.184	
Low FEF_25−75_ (< 75% predicted)			
Low exposure	Ref	–	0.026
Moderate exposure	0.68 (0.13, 3.34)	0.635	
High exposure	1.68 (0.73, 3.90)	0.225	
Low FEV_1_/FVC (< 85%)			
Low exposure	Ref	–	0.121
Moderate exposure	1.56 (0.48, 5.08)	0.457	
High exposure	1.64 (0.86, 3.13)	0.131	

Estimates are difference for continuous outcomes and odds ratio for binary outcomes.

a Adjusted for age, sex, height, weight, secondhand smoke exposure, physical activity, vegetable and fruit intake, parental education, and mean PM_2.5_ concentration on the lung function examination day and 1 day before examination (lag01).

**Figure 1:**
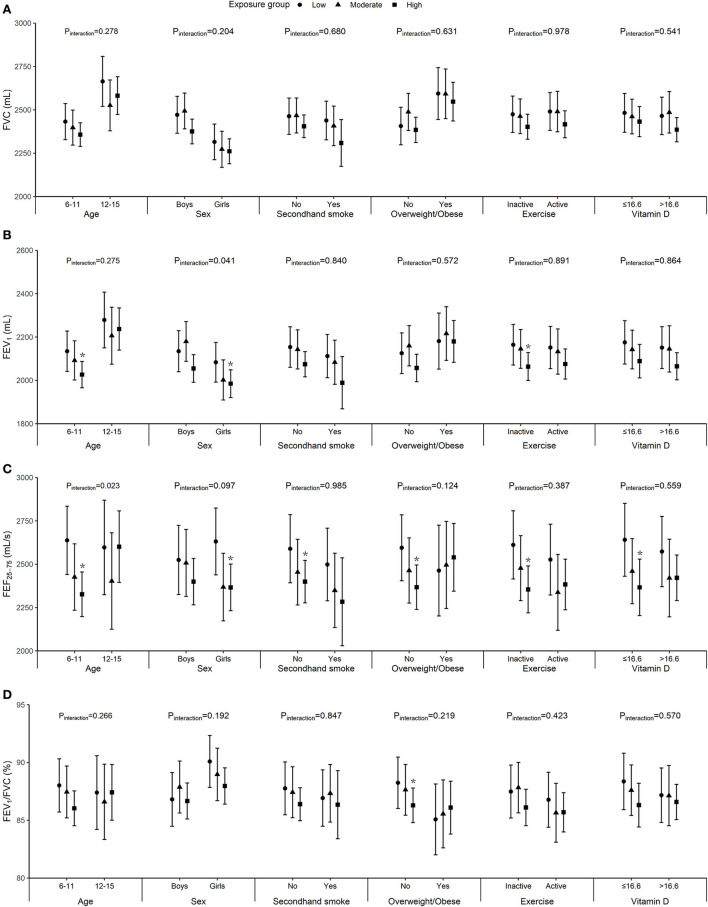
Marginally adjusted means of FVC **(A)**, FEV_1_ **(B)**, FEF_25−75_ **(C)**, and FEV_1_/FVC **(D)** by age, sex, secondhand smoke exposure, body mass index (BMI), physical exercise, and vitamin D (the median value was 16.6 ng/mL). Models were adjusted for age, height, weight, vegetable and fruit intake, parental education, and mean PM_2.5_ concentration on the lung function examination day and 1 day before examination (lag01), and sex, secondhand smoke exposure, and physical exercise when appropriate. Error bars represent 95% CI and asterisk indicates *p* < 0.05.

**Figure 2:**
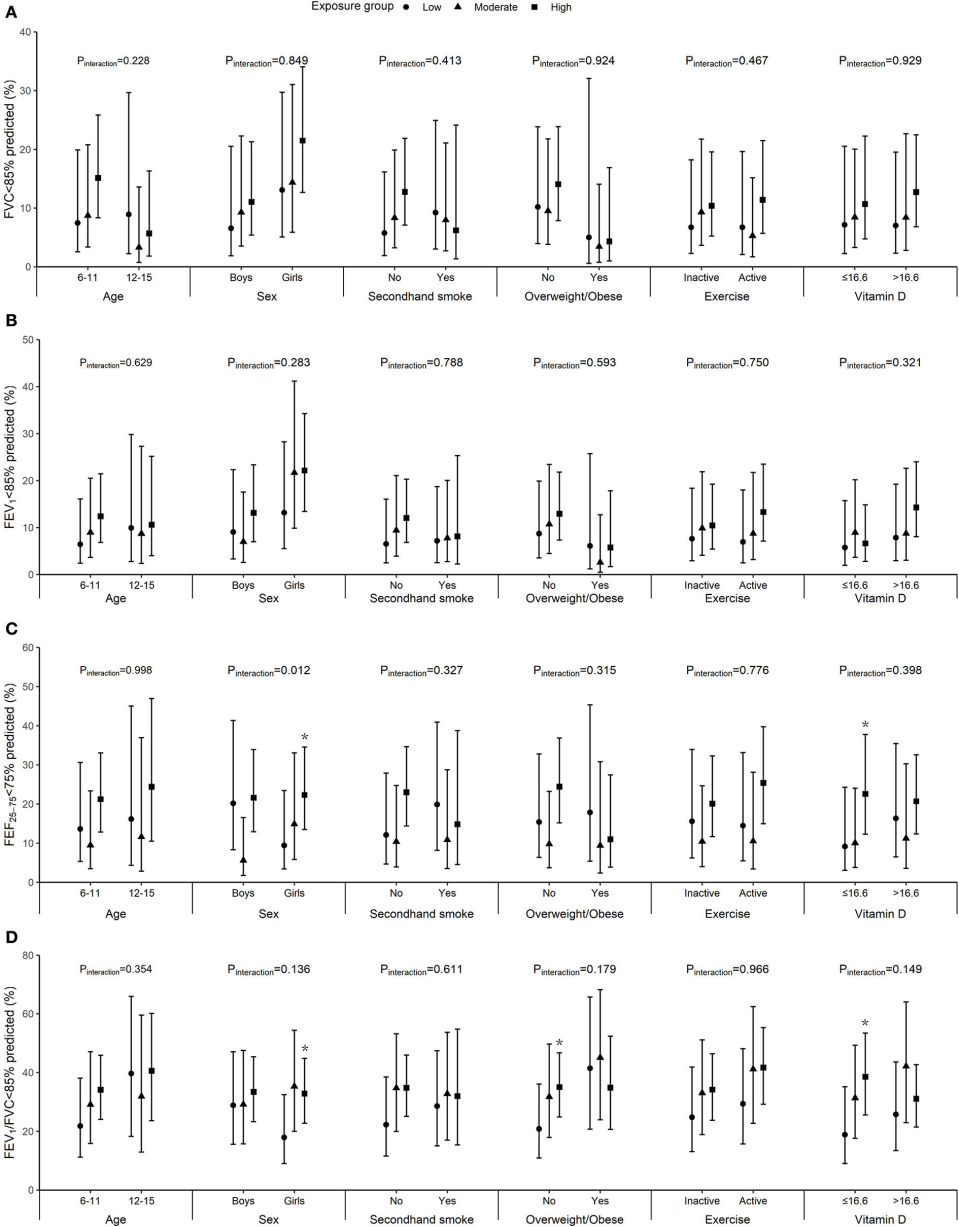
Marginally adjusted probability of low FVC **(A)**, FEV_1_ **(B)**, FEF_25−75_ **(C)**, and FEV_1_/FVC **(D)** by age, sex, secondhand smoke exposure, body mass index (BMI), physical exercise, and vitamin D (the median value was 16.6 ng/mL). Models were adjusted for age, height, weight, vegetable and fruit intake, parental education, and mean PM_2.5_ concentration on the lung function examination day and 1 day before examination (lag01), and sex, secondhand smoke exposure, and physical exercise when appropriate. Error bars represent 95% CI and asterisk indicates *p* < 0.05.

Significant interactions of age and sex with air pollution were observed in relation to FEF_25−75_ and FEV_1_ (p_interaction_ = 0.023 and 0.041, respectively). In addition, although no significant modification effects were found for secondhand smoke exposure, BMI, physical activity, and serum vitamin D levels, we did observe significant associations among children with normal weight, non-regular exercise, lower vitamin D, or without exposure to secondhand smoke but not their counterparts (Figure 1). The differences and 95% CI comparing high vs. low exposure group were −107.9 (95% CI: −195.6, −20.3) mL for FEV_1_ and −311.1 (95% CI: −496.6, −125.5) mL/s for FEF_25−75_ among children aged 11 years or younger, −98.6 (95% CI: −191.7, −5.4) mL for FEV_1_ and −264.9 (95% CI: −461.1, −68.8) mL/s for FEF_25−75_ among girls, −189.7 (95% CI: −366.9, −12.4) mL/s for FEF_25−75_ among children not exposed to secondhand smoke, −227.3 (95% CI: −397.5, −57.2) mL/s for FEF_25−75_ among children with normal weight, −100.6 (95% CI: −196.5, −4.8) mL for FEV_1_ and −256.7 (95% CI: −457.9, −55.6) mL/s for FEF_25−75_ among physically inactive children, and −274.1 (95% CI: −487.7, −60.5) mL/s for FEF_25−75_ among children with lower vitamin D levels. Besides, stronger associations with the prevalence of poor lung function for FEF_25−75_ and/or FEV_1_/FVC were found in girls and children with normal weight or lower vitamin D (Figure 2).

**Figure 3:**
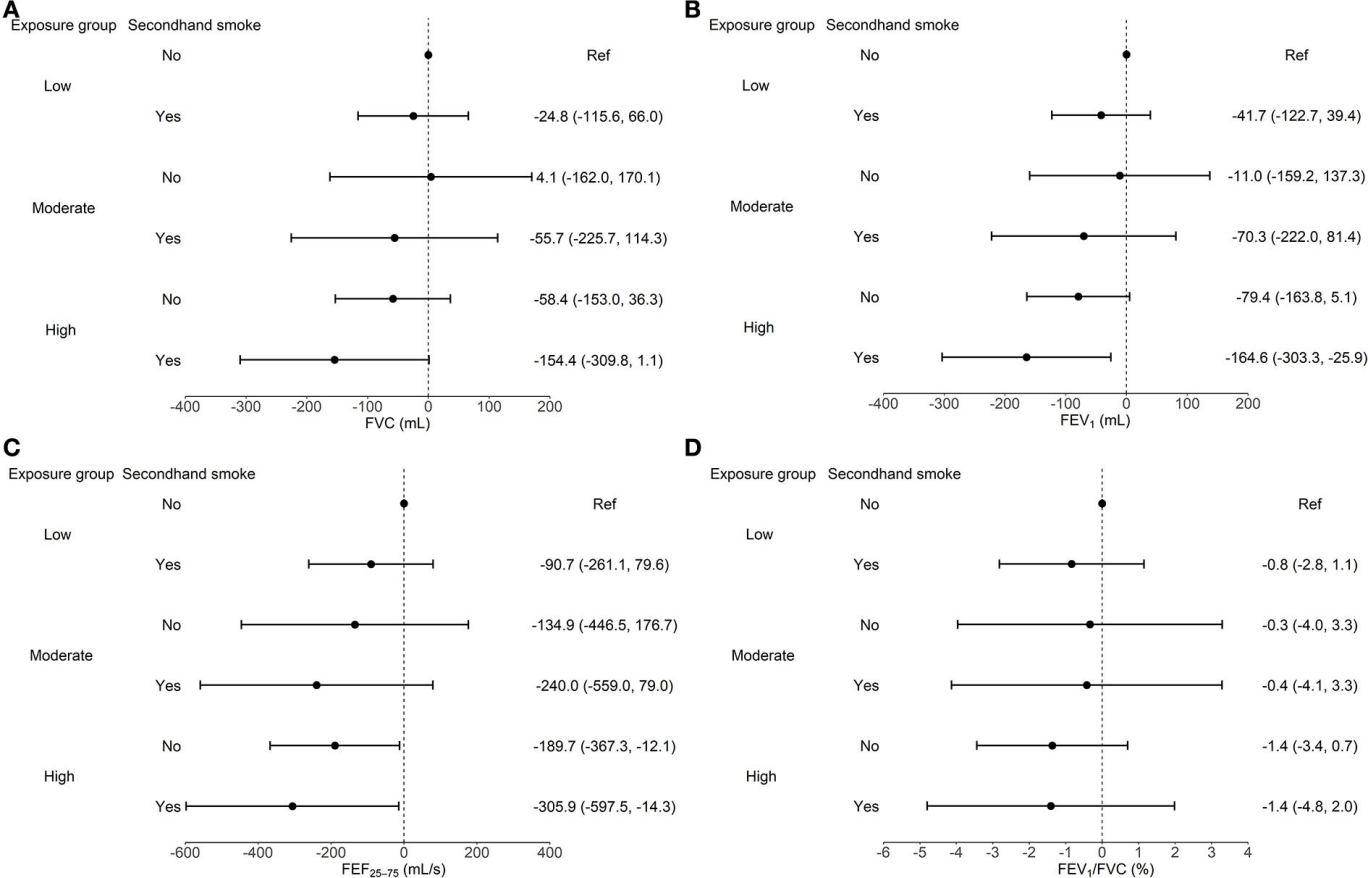
Joint analysis of air pollution and secondhand smoke exposure on FVC **(A)**, FEV_1_ **(B)**, FEF_25−75_ **(C)**, and FEV_1_/FVC **(D)** among all schoolchildren. Models were adjusted for age, sex, height, weight, physical activity, vegetable and fruit intake, parental education, and mean PM_2.5_ concentration on the lung function examination day and 1 day before examination (lag01). Error bars represent 95% CI.

The combined impacts of air pollution and secondhand smoke are shown in Figure 3. Compared with children in the low exposure group and not exposed to secondhand smoke, those exposed to both high level air pollution and secondhand smoke had the worst lung function, with 154.4 (95% CI: −1.1, 309.8) mL decrement in FVC, 164.6 (95% CI: 25.9, 303.3) mL decrement in FEV_1_, and 305.9 (95% CI: 14.3, 597.5) mL/s decrement in FEF_25−75_. Similar analyses related to the prevalence of poor lung function yielded no significant results (data not shown).

In the sensitivity analyses, the inverse trends persisted with regard to associations with percent predicted FEV_1_ and FEF_25−75_ (Supplementary Table S1). Regression results restricting to children not exposed to secondhand smoke or without self-reported asthma and additionally adjusting for short-term NO_2_ or O_3_ did not differ substantially from those based on the whole population (Supplementary Table S2).

## Discussion

To our knowledge, this work represents one of the few studies that investigated the impact of long-term air pollution at contemporary exposure levels in developing countries on the lung function of children in school. Our results showed reduced FEV_1_ and FEF_25−75_ with increasing levels of air pollution. The negative associations were more pronounced among children who were younger, girls, not exposed to secondhand smoke, non-overweight, physically inactive, or vitamin D deficient. Moreover, air pollution and secondhand smoke exhibited joint effects on lung function decline.

Among lung function parameters, the largest change associated with air pollution was found for FEF_25−75_. The same pattern has been observed in regions with low (U.S., Netherlands, Norway, and Italy) and moderate (Hong Kong, China) air pollution level (18–22). Since FEF_25−75_ is more reflective of small airway dysfunction, our finding suggested that air pollution might exert more influence on small airways than on large airways. This is plausible because higher doses of air pollutants are supposed to deposit in the small airways (23). Although we could not disentangle the individual contribution of each pollutant, PM_2.5_ and O_3_ seemed to be responsible for FEF_25−75_ reduction considering their concentration contrasts between cities and the potential of entering into small airways (18, 24). Smaller particles can more easily penetrate into the lower respiratory tract, and a number of studies support the relation between long-term PM_2.5_ exposure and lower FEF_25−75_ (18, 24, 25). Due to relatively low water solubility, O_3_ can also reach and subsequently be retained in small airways, in spite of the epidemiological evidence regarding its effect on FEF_25−75_ are somewhat inconsistent (26, 27). In addition to airway flow measure, parameters indicative of mechanical ventilatory function were also involved here and the negative association was found with FEV_1_ but not FVC. This is in line with many studies reporting a larger association magnitude for FEV_1_ than for FVC (18–22), which indicates a greater impact on airway obstruction than lung size. The observed changes in lung function, although small from the individual perspective, may lead to a shift in population distribution. We therefore further determined the prevalence of lung function parameters below clinically relevant thresholds and found null associations. In contrast, prior studies based on data from Chinese children reported significant effects of chronic air pollution on the prevalence of poor lung function (6, 28). We acknowledged this study might be underpowered to detect such associations. Differences in age range, air pollution level, and formula used to calculate percent predicted values might also help explain the inconsistent results across studies.

Previous studies have established the exposure-response relationship between long-term air pollution exposure and respiratory disease mortality (29, 30). As lung function is a marker of early lung injury, it is interesting to explore the corresponding relationship using lung function as outcome. Nevertheless, the existing evidence is limited, especially for children, and mainly derived from statistical models. A large longitudinal study in China revealed preliminary results that the decreases of lung function appeared to be faster up to PM_2.5_ concentration range of 20–25 μg/m^3^ and slower thereafter (28). In the current study, we provided direct evidence that there is a linear exposure-response relationship between long-term air pollution and lung function impairment when PM_2.5_ is in the range of 25–60 μg/m^3^, which is valuable for assessing the health risk of children in developing countries. Likewise, Tsui et al. (27) found that lifetime exposure to intermediate levels of PM_10_ (25–85 μg/m^3^) was linearly associated with lower lung function in non-asthmatic children from Taiwan, China. In addition, the fact that lung function started to decrease in the moderate exposure group whose exposure level was slightly above interim target 1 (IT-1) values recommended by WHO highlights the necessity of mandating IT-1 standard in countries with moderate and high air pollution to protect vulnerable population.

It is known that the behaviors and lung development of younger children are different from those of older children, leading us to hypothesize that air pollution might display distinct impacts on children of various ages. However, this topic is understudied before possibly due to the narrow age range of the study population. Two cross-sectional studies in Rome and U.S. showed stronger associations of NO_2_ and blood manganese with lung function in older children (21, 31). They speculated that longer cumulative exposure to low-dose air pollution might account for this observation. Unlike these studies, younger children seemed to be more vulnerable in this study, suggesting that the role of cumulative exposure in affecting lung function is less obvious in relatively high pollution settings. Indeed, older children generally have higher antioxidant capacity than younger children, enabling them better protection against air pollution-related oxidative stress. A Japanese study found a positive correlation between the serum level of antioxidant capacity biomarker and age in 77 children (32). Besides, the adaptive responses may be more evident among older children as they are exposed to air pollution for a longer period. Relative to age, researches on sex differences in lung function associated with air pollution are more abundant, but the results are rather mixed. While some studies demonstrated a larger reduction of lung function among girls as seen in this study (19–21), others found opposite patterns or no significant results (22, 33–35). The age more or less close to puberty of our subjects lend us credence that the observed finding may be partially attributed to estrogen, since estrogen level correlates with symptom severity of asthma (36).

Exposure to secondhand smoke and overweight/obesity are common among children, both of which appear to affect lung function. Here, the negative associations were stronger in children not exposed to secondhand smoke. To support this finding, many previous cohorts including Framingham Heart, Normative aging, and UK Biobank studies reported higher effects on never/former smokers for associations between PM_2.5_ and lung function parameters (15, 37, 38). This is not surprising because smoking may have already damaged lung function through inflammation, thus masking the additional effect of air pollution to some extent. However, this does not necessarily imply that avoidance of exposure to secondhand smoke is meaningless for children. Notably, the mean levels of FVC, FEV_1_, and FEF_25−75_ were all lower in children exposed to secondhand smoke, and the largest reduction of lung function parameters was found in children who lived in the most polluted area and were exposed to secondhand smoke. As a result, campaigns protecting children from secondhand smoke should be advocated as ever. On the other hand, we found children with normal weight might be more sensitive to the respiratory effect of air pollution, which could also be explained by the pre-existing inflammation induced by excess fat. Contradicting result was reported by the Seven Northeastern Chinese Cities study (6). In that study, the associations between prevalence of lung function impairment and air pollutants were strongest in obese children, followed by overweight children, and weakest in normal-weight children. The modification of age on the relationship of BMI with lung function is likely to shed some light on the discrepancy. In younger children, lung function tends to be higher with increasing BMI (39). But after puberty, their relationship is characterized by an inverted U shape, with lower lung function at both ends of BMI (40). Given our population composition (about 75% are from primary school), the detrimental effect of overweight/obesity on lung function is not expected. This postulation is further evidenced by the stratified result that the modification of overweight/obesity on the association between particulate matter and lung function impairment was more evident in older children than in younger children (6).

As aforementioned, children take physical activity more regularly than adults and higher physical activity has a favorable effect on lung function. Meanwhile, more air pollutants are inhaled by children during exercise. It remains equivocal on whether physical activity promotes or attenuates the adverse health effects of air pollution, which is also a major concern for many parents and policy makers. In the present study, we found physically inactive children might be more susceptible than active children. Similarly, a Chinese nationwide study found the impact of PM_10_ on FVC was stronger in inactive children (7). Both results add evidence for guiding the behavior of children during pollution days. Although biological mechanisms underlying the modification effect of physical activity are not fully understood, anti-inflammation should be one of the possible pathways. Olivo et al. (41) found that mice undergoing 10-week exercise training (5 days/week, 1 hour/day) exhibited lower proinflammatory cytokines in the lung in response to diesel exhaust particle exposure.

Micronutrients are indispensable for the maintenance of normal tissue function and their deficiencies can cause deficits in lung function (42). Previous studies have explored the role of micronutrients, especially those with antioxidant properties, in modifying the association between air pollution and lung function. For instance, Romieu et al. (43) observed a significantly negative association of O_3_ at 1 day before spirometry with FEV_1_ and FEF_25−75_ in children with asthma receiving a placebo but not those receiving vitamin supplement (vitamin C and E). Although vitamin D is well known for its implication in calcium homeostasis, there is growing evidence relating vitamin D to lung function and asthma (44, 45). In Normative aging study, smokers with vitamin D deficiency were found to have lower lung function and more rapid lung function decline (46). To our knowledge, this is the first study demonstrating that a lower level of vitamin D might aggravate the reduction of lung function among healthy children exposed to long-term air pollution. Animal study manifested mice exposed to vitamin D deficiency had increased airway smooth muscle mass, which is a feature of airway remodeling of asthma and chronic obstructive pulmonary disease (47). Given the cross-sectional nature of our study, however, whether vitamin D supplements can alleviate the harmful effects of air pollution on the respiratory system needs further investigations using a well-designed randomized controlled trial.

This study has several limitations. First, air pollution data of fixed monitoring stations were used as a proxy of children's exposure level as detailed residential address was not mandatory in the questionnaire, raising concerns of exposure misclassification. However, such measurement errors should not be substantial in terms of characterizing long-term exposure and non-differential misclassification usually biases estimates toward no association. Second, although we did find an increased prevalence of poor lung function in the high exposure group as well as stronger associations in certain subgroups, most of these results did not reach statistical significance possibly owing to sample size. Third, only including vitamin D is not adequate to provide a full picture of nutritional intervention in alleviating the adverse health effects of air pollution. Fourth, parental education was severely imbalanced across exposure groups. Covariate adjustment might not fully remove residual confounding. Finally, cotinine was not determined to measure the actual exposure level of secondhand smoke more accurately, nor moderate and vigorous physical activities were differentiated from each other.

In conclusion, our findings indicate that long-term air pollution at levels of exposure currently experienced by many people in developing countries is associated with lower levels of large and small airway function in Chinese children, with a stronger impact on the latter. Reducing secondhand smoke exposure, encouraging physical activity, and supplementing vitamin D are important public health priorities to mitigate the burden of respiratory disease among children in China and other developing countries with comparable air pollution levels. Future studies measuring individual exposure and analyzing the data with more advanced methods (e.g., exposome-wide association) are warranted to provide more insight into the impact of long-term air pollution on lung growth of children and the potential effect of air pollution regulation policies (48, 49).

## Data availability statement

The raw data supporting the conclusions of this article will be made available by the authors, without undue reservation.

## Ethics statement

The studies involving human participants were reviewed and approved by Research Ethics Committee of the Anhui Center for Disease Control and Prevention and the National Institute for Occupational Health and Poison Control, Chinese Center for Disease Control and Prevention. Written informed consent to participate in this study was provided by the participants' legal guardian/next of kin.

## Author contributions

WL, HD, and JT contributed to conceptualization and critical revision of the manuscript. JT collected and interpreted the data. JL conducted data analysis and drafted the manuscript. All authors contributed to the article and approved the submitted version.
