# Supplementary material for: Long-term exposure to air pollution and lung function among children in China: Association and effect modification
Source: Front Public Health. 2022 Dec 14;10:988242. doi: 10.3389/fpubh.2022.988242 (PMC9795025; doi:10.3389/fpubh.2022.988242)
Supplement: Supplementary file 1 [file Data_Sheet_1.docx]

Table S1 Multivariable adjusted^a^ associations between air pollution levels and percent predicted lung function parameters among all schoolchildren

|  | Difference (95% CI) | P value^a^ | P_trend_^a^ |
| --- | --- | --- | --- |
| Percent predicted FVC |  |  |  |
| Low exposure | Ref | - | 0.209 |
| Moderate exposure | 4.1 (-2.0, 10.2) | 0.185 |  |
| High exposure | -0.5 (-3.9, 3.0) | 0.791 |  |
| Percent predicted FEV_1_ |  |  |  |
| Low exposure | Ref | - | 0.046 |
| Moderate exposure | 2.0 (-4.1, 8.1) | 0.524 |  |
| High exposure | -2.1 (-5.5, 1.4) | 0.242 |  |
| Percent predicted FEF_25-75_ |  |  |  |
| Low exposure | Ref | - | 0.036 |
| Moderate exposure | -3.3 (-14.7, 8.2) | 0.577 |  |
| High exposure | -6.1 (-12.6, 0.4) | 0.065 |  |
| Percent predicted FEV_1_/FVC |  |  |  |
| Low exposure | Ref | - | 0.099 |
| Moderate exposure group | -2.3 (-6.0, 1.5) | 0.234 |  |
| High exposure | -2.0 (-4.1, 0.1) | 0.067 |  |

^a^Adjusted for weight, secondhand smoke exposure, physical activity, vegetable and fruit intake, parental education, and mean PM_2.5_ concentration on the lung function examination day and 1 day before examination (lag01).

Table S2 Sensitivity analyses of associations between air pollution levels and lung function parameters among schoolchildren

|  | **Exposure group** | | | P_trend_ |
| --- | --- | --- | --- | --- |
|  | Low | Moderate | High |  |
| **Without Secondhand smoke exposure^a^** |  |  |  |  |
| FVC | Ref | -6.7 (-200.3, 187.0) | -50.3 (-154.1, 53.6) | 0.197 |
| FEV_1_ | Ref | -26.8 (-201.6, 148.1) | -79.1 (-172.9, 14.6) | 0.036 |
| FEF_25-75_ | Ref | -177.6 (-551.1, 195.8) | -203.5 (-403.7, -3.2) | 0.038 |
| FEV_1_/FVC | Ref | -0.3 (-4.5, 3.9) | -1.8 (-4.1, 0.5) | 0.037 |
| Low FVC (<85% predicted) | Ref | 1.44 (0.21, 9.86) | 1.68 (0.55, 5.40) | 0.322 |
| Low FEV_1_ (<85% predicted) | Ref | 1.84 (0.33, 10.04) | 1.83 (0.71, 4.82) | 0.226 |
| Low FEF_25-75_ (<75% predicted) | Ref | 0.92 (0.13, 6.36) | 2.40 (0.90, 6.59) | 0.007 |
| Low FEV_1_/FVC (<85%) | Ref | 1.99 (0.47, 8.41) | 1.98 (0.94, 4.22) | 0.072 |
| **Without self-reported diagnosis of asthma^b^** |  |  |  |  |
| FVC | Ref | -13.6 (-171.3, 144.0) | -77.3 (-165.5, 10.9) | 0.025 |
| FEV_1_ | Ref | -21.0 (-161.7, 119.7) | -91.7 (-170.5, -12.9) | 0.003 |
| FEF_25-75_ | Ref | -132.6 (-429.0, 163.8) | -198.5 (-364.4, -32.5) | 0.010 |
| FEV_1_/FVC | Ref | 0.0 (-3.4, 3.5) | -1.2 (-3.1, 0.7) | 0.095 |
| Low FVC (<85% predicted) | Ref | 1.37 (0.28, 6.55) | 1.91 (0.75, 5.03) | 0.114 |
| Low FEV_1_ (<85% predicted) | Ref | 1.29 (0.30, 5.42) | 1.71 (0.76, 3.92) | 0.128 |
| Low FEF_25-75_ (<75% predicted) | Ref | 0.63 (0.12, 3.19) | 1.67 (0.73, 3.93) | 0.023 |
| Low FEV_1_/FVC (<85%) | Ref | 1.60 (0.48, 5.27) | 1.69 (0.88, 3.24) | 0.104 |
| **Additionally adjusted for NO_2_ at lag01 day^b^** |  |  |  |  |
| FVC | Ref | -39.7 (-208.7, 129.4) | -69.6 (-158.2, 19.0) | 0.070 |
| FEV_1_ | Ref | -34.2 (-185.3, 116.9) | -79.2 (-158.4, 0.0) | 0.015 |
| FEF_25-75_ | Ref | -157.7 (-475.0, 159.6) | -176.1 (-342.4, -9.8) | 0.031 |
| FEV_1_/FVC | Ref | -0.4 (-4.1, 3.3) | -1.2 (-3.1, 0.8) | 0.135 |
| Low FVC (<85% predicted) | Ref | 0.72 (0.12, 3.84) | 1.57 (0.63, 4.02) | 0.099 |
| Low FEV_1_ (<85% predicted) | Ref | 0.91 (0.18, 4.30) | 1.61 (0.71, 3.70) | 0.085 |
| Low FEF_25-75_ (<75% predicted) | Ref | 0.41 (0.05, 2.54) | 1.42 (0.60, 3.34) | 0.027 |
| Low FEV_1_/FVC (<85%) | Ref | 1.27 (0.34, 4.58) | 1.51 (0.79, 2.90) | 0.129 |
| **Additionally adjusted for O_3_ at lag01 day^b^** |  |  |  |  |
| FVC | Ref | -11.8 (-175.4, 151.8) | -58.3 (-149.9, 33.2) | 0.159 |
| FEV_1_ | Ref | -14.8 (-160.9, 131.4) | -75.2 (-157.1, 6.6) | 0.042 |
| FEF_25-75_ | Ref | -132.5 (-439.4, 174.3) | -174.9 (-346.7, -3.2) | 0.045 |
| FEV_1_/FVC | Ref | -0.1 (-3.6, 3.5) | -1.0 (-3.0, 1.0) | 0.235 |
| Low FVC (<85% predicted) | Ref | 0.88 (0.17, 4.38) | 2.13 (0.84, 5.49) | 0.053 |
| Low FEV_1_ (<85% predicted) | Ref | 1.17 (0.26, 5.18) | 1.96 (0.84, 4.60) | 0.082 |
| Low FEF_25-75_ (<75% predicted) | Ref | 0.63 (0.12, 3.26) | 1.67 (0.70, 4.02) | 0.090 |
| Low FEV_1_/FVC (<85%) | Ref | 1.54 (0.45, 5.23) | 1.61 (0.83, 3.16) | 0.171 |

Estimates are difference for continuous outcomes and odds ratio for binary outcomes.

^a^Models were adjusted for age, sex, height, weight, physical activity, vegetable and fruit intake, parental education, and mean PM_2.5_ concentration on the lung function examination day and 1 day before examination (lag01); ^b^Models were adjusted for age, sex, height, weight, secondhand smoke exposure, physical activity, vegetable and fruit intake, parental education, and mean PM_2.5_ concentration on the lung function examination day and 1 day before examination (lag01).


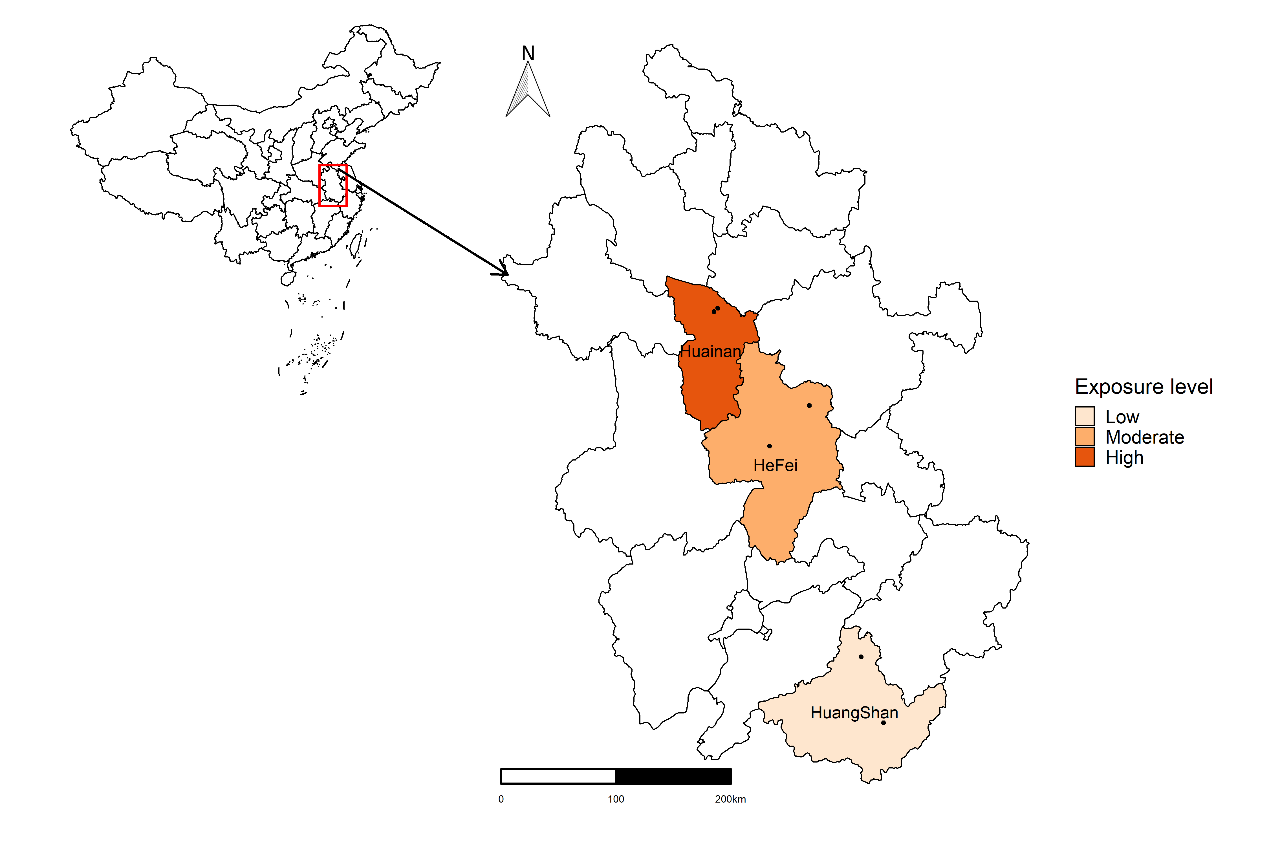


Figure S1 The locations of study sites. The black dots represent corresponding schools within the site.
